# Photoluminescence of Chemically and Electrically Doped Two-Dimensional Monolayer Semiconductors

**DOI:** 10.3390/ma17163962

**Published:** 2024-08-09

**Authors:** Hyungjin Kim, Valerio Adinolfi, Sin-Hyung Lee

**Affiliations:** 1 Department of Materials Science and Engineering, Yonsei University, Seoul 03722, Republic of Korea; 2 Department of Electrical Engineering and Computer Sciences, University of California, Berkeley, CA 94720, USA; ziofitt@gmail.com; 3 Department of Intelligent Semiconductor Engineering, School of Advanced Fusion Studies, University of Seoul, Seoul 02504, Republic of Korea

**Keywords:** photoluminescence, doping, 2D semiconductor, monolayer, TMDC

## Abstract

Two-dimensional (2D) transition metal dichalcogenide (TMDC) monolayers exhibit unique physical properties, such as self-terminating surfaces, a direct bandgap, and near-unity photoluminescence (PL) quantum yield (QY), which make them attractive for electronic and optoelectronic applications. Surface charge transfer has been widely used as a technique to control the concentration of free charge in 2D semiconductors, but its estimation and the impact on the optoelectronic properties of the material remain a challenge. In this work, we investigate the optical properties of a WS_2_ monolayer under three different doping approaches: benzyl viologen (BV), potassium (K), and electrostatic doping. Owing to the excitonic nature of 2D TMDC monolayers, the PL of the doped WS_2_ monolayer exhibits redshift and a decrease in intensity, which is evidenced by the increase in trion population. The electron concentrations of $3.79\times 10^{13}\;{\mathrm{cm}}^{-2}$, $6.21\times 10^{13}\;{\mathrm{cm}}^{-2}$, and $3.12\times 10^{12}\;{\mathrm{cm}}^{-2}$ were measured for WS_2_ monolayers doped with BV, K, and electrostatic doping, respectively. PL offers a direct and versatile approach to probe the doping effect, allowing for the measurement of carrier concentration in 2D monolayer semiconductors.

## 1. Introduction

Two-dimensional (2D) semiconducting material has attracted great attention over recent years. Its unique properties including self-terminated surfaces, capability of forming van der Waals heterostructures, and layer number-dependent electrical and optical characteristics enabled 2D semiconductors as candidates to be applicable for next-generation optoelectronic and electronic devices [1,2,3,4,5]. In particular, a transition metal dichalcogenide (TMDC), a representative type of 2D semiconducting materials such as MoS_2_, WS_2_, and WSe_2_, exhibits an indirect-to-direct bandgap transition at a monolayer thickness level [6,7,8]. Despite its peculiar properties, its widespread applications for practical devices have been limited due to a lack of a reliable and controllable doping technique and approach to evaluate the precise doping concentrations and distributions, which are prevalently implemented in conventional complementary metal–oxide–semiconductor (CMOS) technology. 

The types of doping strategy for 2D monolayer semiconductors include substitution of atoms, creation of vacancy defects, and surface charge transfer [9,10,11]. Substitutional doping is performed by replacing the transition metal or chalcogenide atoms in a monolayer semiconductor and tuning the composition between different elements. Although the doping effect can persist as long as the elemental composition is maintained, it is challenging to achieve desirable doping concentration and distribution during synthesis. Unlike in bulk semiconductors, the bombardment of high energy ions onto 2D monolayer semiconductors not only induces doping via vacancy creation but also impairs the materials, thus degrading the performance of monolayer semiconductors because of their atomically thin nature [12]. Surface charge transfer, on the other hand, induces the doping effect without structural or chemical changes in a monolayer semiconductor. With physical or chemical adsorption of the layer applied, charge carriers migrate from the deposited layer to the adjacent material by diffusion [13,14,15]. Moreover, the electrostatic field can drive the transport of charge carriers by drift. There have been many approaches and studies on surface charge transfer reported as accessible and efficient methods of doping for 2D monolayer semiconductors [16,17,18,19,20]. However, the surface charge transfer method still involves bottlenecks in the sense that it lacks controllability. In fact, it is demanding to quantify the amount of charge carriers that are transferred to the 2D monolayer semiconductor. Therefore, a systemic approach for determining the effectiveness of surface charge transfer doping on 2D monolayer semiconductors needs to be developed for their practical application, but no such directions have been studied so far. 

Recently, recombination physics in 2D TMDC monolayer semiconductors has been explored, which is largely distinct from bulk semiconductors owing to the reduced Coulomb interactions [21,22,23]. Photoluminescence (PL) quantum yield (QY) is a parameter that is calculated as a ratio of the number of photons emitted to the number of photons absorbed in a material, which is also decided with a relative rate between radiative recombination and nonradiative recombination. PL QY is of significance as a key metric for optoelectronic applications since it directly determines the ultimate efficiency limit that the device can achieve. TMDC monolayers, however, suffer from poor PL QY at room temperature. A number of strategies have been proposed to enhance the low PL QY of TMDC monolayer semiconductors, leading to the demonstration of a near-unity PL QY in monolayer MoS_2_ at low exciton generation rates [24,25,26]. Since neutral exciton recombination is entirely radiative even at high defect densities, the photophysics of 2D TMDC monolayer semiconductors is dictated by the relative population of neutral excitons and trions, which are formed from excitons interacting with background charge carriers [27,28,29]. 

In this work, the optical properties of a doped 2D monolayer semiconductor were investigated. Since free carriers form trions to have nonradiative recombination, PL QY of the doped monolayer semiconductor at a certain exciton generation rate offers information on the population of excitons and trions, which the carrier concentration can be extracted from. We further leverage the recombination model of a 2D TMDC monolayer semiconductor to compare and analyze the effect of two surface charge transfer methods: chemical and electrostatic doping. Without perturbating the characteristics of doped 2D monolayer semiconductors, PL promptly reflects the results of doping applied in a monolayer semiconductor. Moreover, it serves as an efficient probe to estimate a wide range of carrier concentration-dependent optical property variations. Our study shows the potential of PL to be employed for characterizing semiconducting materials with various doping conditions even with high defect density.

## 2. Materials and Methods

WS_2_ was mechanically exfoliated from a single crystal source (HQ Graphene, Groningen, The Netherlands) onto a 50 nm SiO_2_/p+-Si substrate. Then, monolayers were identified under microscopes with their optical contrast. For a back-gated field-effect transistor (FET), source and drain contacts were patterned via electron beam (e-beam) lithography using PMMA (C4, MicroChem, Austin, TX, USA) as an e-beam resist followed by the e-beam evaporation of Ti/Au (5/25 nm) for electrodes. Electrical measurements were performed with the B1500A semiconductor device parameter analyzer (Keysight, Santa Rosa, CA, USA).

Benzyl viologen (BV) molecules were prepared as a solution for chemical n-doping. Starting with benzyl viologen dichloride (20 mg, Sigma-Aldrich, St. Louis, MO, USA) dissolved in deionized (DI) water and toluene (5 mL/5 mL), the solution was kept for one day after adding sodium borohydride (4 g, Sigma-Aldrich). Doping was performed by extracting and drop-casting the upper layer (toluene) of the bilayer solution onto the sample, followed by N_2_ gas to remove the excess molecules and solvents under ambient conditions. K doping was carried out under vacuum conditions (~5 × 10^−5^ Torr) inside a home-built chamber where a 5 A current flows through a filament to heat up a boat and K vapor is generated to be evaporated onto the sample for a controlled exposure time. For K doping, a minimum time of 250 s is required to vaporize the K dopants. 

Optical measurements were performed in a customized micro-PL setup under ambient conditions (298–300 K, ~20–30% relative humidity). A laser diode with a 532 nm peak emission wavelength (CNI Laser, Changchun, China) was used as an excitation source and the PL signal was collected with a 50× (NA = 0.55) objective lens (Olympus, Tokyo, Japan) and sent to a spectrometer (DXG) and CCD detector (iDus 420 BEX2-DD, Andor, Abingdon, UK). The detailed calibration procedure of the setup to extract PL QY is provided in previous work [24]. A reference sample (Rhodamine 101, Sigma-Aldrich) of which the PL QY is known was used to confirm the extracted PL QY value and cross-calibrate our setup. For time-resolved PL spectroscopy, the sample was excited by a picosecond (10–20 ps pulse duration) 48 MHz pulsed laser with a 532 nm peak emission wavelength (CNI Laser). The PL signal was detected using a single photon avalanche diode (PDM-50, MPD, Bolzano, Italy) and the time-correlated single photon counting (TCSPC) module (PicoHarp 300, PicoQuant, Berlin, Germany) acquired the synchronized PL decay to deduce the lifetime. Micro-absorption spectroscopy was performed by obtaining micro-reflection and micro-transmission spectra from the sample using a supercontinuum laser (FIU-6, NKT Photonics, Birkerod, Denmark).

Top-gated WS_2_ devices were fabricated using a poly(methyl2methacrylate) (PMMA; 950 A11, MicroChem)-assisted pick-and-place dry transfer method. Hexagonal boron nitride (hBN) (HQ Graphene) with ~50~100 nm thickness and monolayer graphene (HQ Graphene) were exfoliated from single crystal sources and used for the gate dielectric and electrodes, respectively. For gate voltage-dependent PL measurement, gate voltages were applied with a source meter (Model 2410, Keithley, Cleveland, OH, USA) through the top graphene electrode, while the WS_2_ monolayer was electrically ground during measurement.

## 3. Results and Discussion

**Figure 1:**
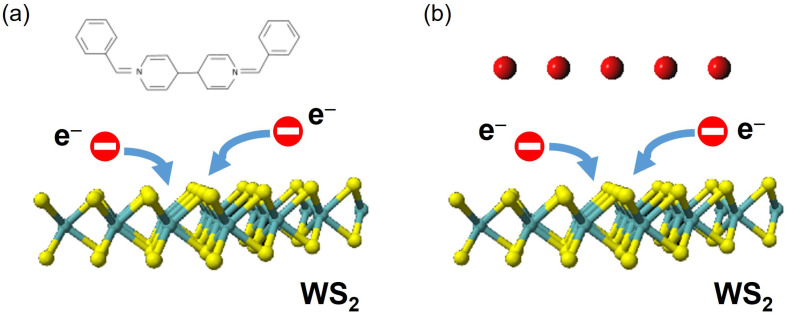
Schematic describing the n-doping procedure of the WS_2_ monolayer with (**a**) a BV molecule and (**b**) K vapor. (Sulfur atoms: yellow, Tungsten atoms: cyan).

We applied two chemical doping methods for surface charge transfer onto the WS_2_ monolayer, which are BV and K doping. Figure 1a illustrates the BV doping process, where the neutral BV molecule donates electrons to an acceptor material, which is monolayer WS_2_ in this case. Since the BV possesses significantly low reduction potentials, n-doping is enabled and maintained under ambient conditions [16]. Figure 1b describes the process of K doping. When the K vapor is deposited onto the WS_2_ monolayer, the small electron affinity of K yields the transfer of electrons from K to WS_2_, achieving n-doping in the monolayer semiconductor.

### 3.1. BV-Doped 2D Monolayer Semiconductor

**Figure 2:**
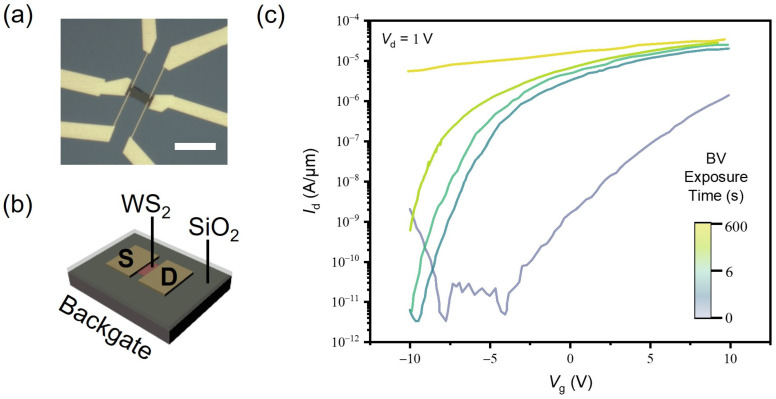
(**a**) Optical microscope images of the WS_2_ monolayer FET. The scale bar is 20 μm. (**b**) Schematic of the FET device structure with a p+-Si backgate electrode and a 50 nm SiO_2_ gate dielectric. (**c**) *I*_d_-*V*_g_ transfer curves of the monolayer WS_2_ FET at $V_{d}=1\;V$ with increasing BV doping times.

In order to estimate the effectiveness of n-doping with BV, we fabricated an FET based on the WS_2_ monolayer and measured its transfer characteristics. Figure 2a shows an optical micrograph of the fabricated WS_2_ monolayer FET and Figure 2b illustrates the device structure where the gate voltage is applied through the backgate of the SiO_2_/p+-Si substrate. In Figure 2c, the *I*_d_-*V*_g_ transfer curves of the monolayer WS_2_ FET are provided with respect to BV doping durations. Here, the device was immersed into the BV solution for controlled times and dried under N_2_ prior to measurement. The as-fabricated monolayer WS_2_ FET exhibits an ambipolar characteristic with both electron and hole conduction, determined by applied gate bias. Upon BV doping, the transfer curves of the device start to change significantly, with its electron conduction drastically increasing with a threshold voltage shift toward a more negative voltage. After 600 s of BV doping, the on-current level increases more than an order of magnitude, and only a small gate control over *I*_d_ is observed, indicating a strong doping effect by BV molecules. The electron concentration after BV doping in the WS_2_ monolayer can be calculated from the equation:

(1) $$n_{2D}=\frac{I_{d}L}{qWV_{d}\mu }$$

where $n_{2D}$ is a carrier density in a 2D sheet, L is the channel length, q is the elementary charge, W is the channel width, and μ is the field-effect mobility. From the transfer curve, we obtained the field-effect mobility of $\mu =26.1\;{\mathrm{cm}}^{2}V^{-1}s^{-1}$ and thus the 2D electron density of $n_{2D}=3.79\times 10^{13}\pm 8.18\times 10^{14}\;{\mathrm{cm}}^{-2}$. 

### 3.2. Potassium-Doped 2D Monolayer Semiconductor

**Figure 3:**
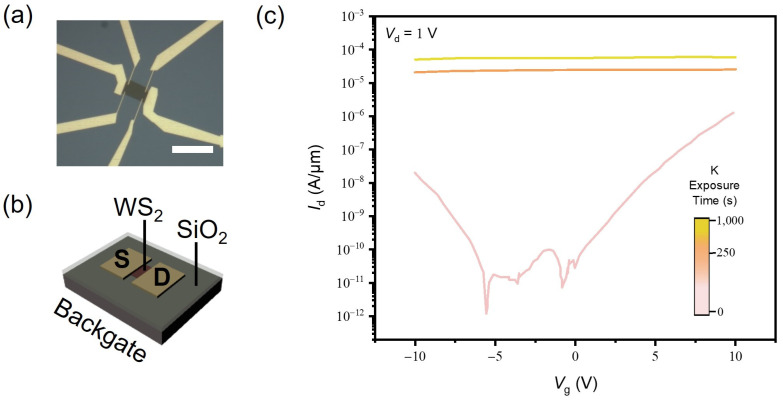
(**a**) Optical microscope images of the WS_2_ monolayer FET. The scale bar is 20 μm. (**b**) Schematic of the FET device structure with a p+-Si backgate electrode and a 50 nm SiO_2_ gate dielectric. (**c**) *I*_d_-*V*_g_ transfer curves of the monolayer WS_2_ FET at $V_{d}=1\;V$ with increasing K doping times.

Doping the WS_2_ monolayer with K results in strong n-type surface charge transfer doping. Owing to its reduction potential of −2.93 V, K doping has been used for improving contact resistance, which often restricts the performance of electronic devices [17]. To evaluate the doping effect with K, we fabricated a backgated FET, as shown in Figure 3a,b. In contrast with BV-doped WS_2_ devices, the devices were measured under vacuum conditions as air exposure diminishes the doping effect due to the oxidation of K. In Figure 3c, it is shown that the *I*_d_-*V*_g_ curve changes drastically as soon as the WS_2_ monolayer is exposed to K vapor, which is why only a small dynamic range of K exposure time was covered in this experiment. After K doping, the *I*_d_ lost gate voltage dependence and the on-current level increased more than orders of magnitude, exhibiting a clear consequence of degenerate n-doping. Similarly to the BV-doped WS_2_ monolayer, the 2D electron density of $n_{2D}=6.21\times 10^{13}\pm 5.74\times 10^{14}\;{\mathrm{cm}}^{-2}$ was calculated, which is higher than the value obtained for the same material but with BV doping and corresponds to the degenerate limit. This high electron concentration after K doping shows that there will be surface-dominant electronic transport, which can vary significantly with the different layer numbers of WS_2_.

### 3.3. Luminescence Properties of the Chemically Doped 2D Monolayer Semiconductor

**Figure 4:**
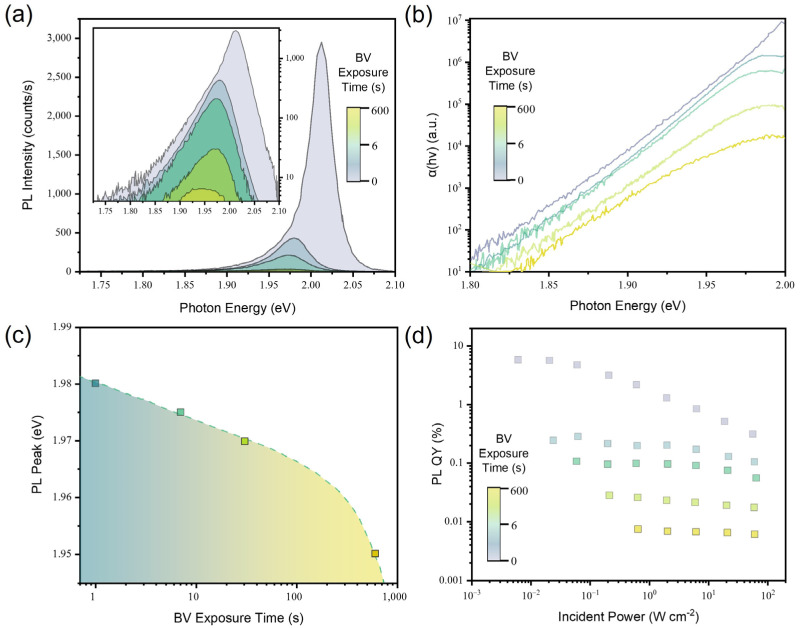
(**a**) PL spectra of the WS_2_ monolayer measured at an incident power of 2 W/cm^2^ with increasing BV exposure time. (**b**) The Urbach tail of the WS_2_ monolayer as a function of BV exposure time. (**c**) The relationship between PL peak position and BV exposure times. (**d**) Incident power-dependent PL QY of the WS_2_ monolayer with increasing BV exposure time.

Next, we characterized the optical properties of BV- and K-doped WS_2_ monolayers. We first analyzed the PL of the WS_2_ monolayer as a function of BV doping time. In Figure 4a, the decreasing behaviors of PL intensity are observed with increasing BV doping durations. The PL intensity decrease with increasing BV doping is attributed to the increase in trion populations in the WS_2_ monolayer. The recombination kinetics in the WS_2_ monolayer is depicted using the following equation:

(2) $$G=\frac{n_{X}}{\tau_{X}}+\frac{n_{T}}{\tau_{T}}+C_{bX}n_{X}^{2}$$

where G is the generation rate of excitons, $n_{X}$ and $n_{T}$ are the neutral exciton and trion concentrations, respectively, $\tau_{X}$ and $\tau_{T}$ are the neutral exciton and trion lifetimes, respectively, and $C_{bX}$ is the biexciton annihilation coefficient [27]. Then, PL QY is determined based on the equation below:

(3) $$PLQY=\frac{1}{G}\left(\frac{n_{X}}{\tau_{X_{r}}}+\frac{n_{T}}{\tau_{T_{r}}}\right)$$

This provides us with theoretical guidance on the generation and recombination rates of quasi-particles consisting of monolayer semiconductors. The relationship between a negative charge concentration (N), trion concentration, and free electron concentration ($n_{e}$) is described as $N=n_{e}+n_{T}$. In particular, when electrons are dominant in a monolayer semiconductor, this relationship further expands to the following equation:

(4) $$n_{T}=\frac{Tn_{X}}{1+Tn_{X}}N$$

where T is the trion formation coefficient. Unless the exciton concentration is extremely high, meaning $Tn_{X}\gg 1$, which is the case when the trion concentration becomes equivalent to the negative charge concentration ($n_{T}\approx N$), free electrons transferred into a monolayer semiconductor by doping contribute to the negative trion formation with excitons. Because the radiative lifetime of trions in the WS_2_ monolayer ($\tau_{T_{r}}$ = ~0.032 μs) is about 300 times longer than the nonradiative lifetime of trions in the WS_2_ monolayer ($\tau_{T_{nr}}$ = ~0.1 ns), this leads to a dramatic increase in the nonradiative recombination rate and thus the PL intensity decreases [27]. Figure 4b presents the Urbach tail of the WS_2_ monolayer with different BV exposure times. The slope of Urbach tails remains the same for increasing BV exposure times, indicating that there is no evidence of additional defect states produced by BV doping. 

The redshift of the PL emission spectra is observed with increasing BV doping times, as shown in Figure 4c. Given that the additional binding energy is required to form a trion from a neutral exciton, this result validates the PL intensity decrease which is associated with the increased trion population in the BV-doped WS_2_ monolayer. Figure 4d shows the PL QY of the WS_2_ monolayer after BV-doping at different amounts of time. We observed a monotonic decrease in the PL QY for all incident power ranges as the BV exposure time became longer, which is attributed to the high nonradiative recombination rates of trions.

**Figure 5:**
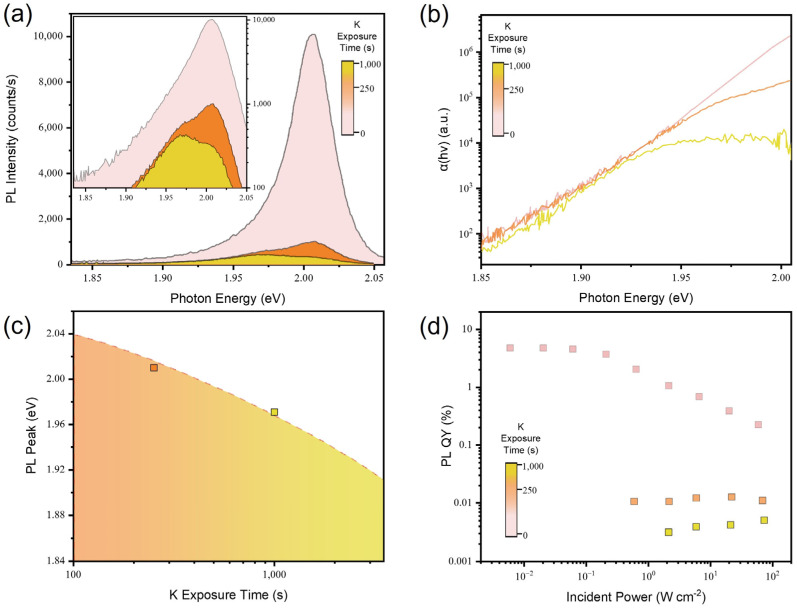
(**a**) PL spectra of the WS_2_ monolayer measured at an incident power of 60 W/cm^2^ with increasing K exposure time. (**b**) The Urbach tail of WS_2_ monolayer as a function of K exposure time. (**c**) The relationship between PL peak position and K exposure time. (**d**) Incident power-dependent PL QY of the WS_2_ monolayer with increasing K exposure time.

Similarly, WS_2_ monolayers with K doping were characterized by their PL emission spectra, as provided in Figure 5a. The same trend of decrease in PL intensity is observed in the WS_2_ monolayer after K doping. In contrast to the BV-doped WS_2_ monolayer, the neutral exciton emission peak remains and coexists with a trion emission peak when K doping is applied. This is possibly a consequence of a change in trion formation coefficient, triggered by strong binding between the K dopant and S plane in the lattice structure of WS_2_ [17]. K doping does not bring about the introduction of defects or disorders which can act as recombination centers. As shown in Figure 5b, the preserved slope of Urbach tails suggests that there is no lattice distortion or change in the density of states near the band edge upon K doping. With the increasing amount of K doping, the PL emission peak is shifted to a lower photon energy, owing to the increase in the trion population, as shown in Figure 5c. In the same way as BV doping, Figure 5d shows the decreased PL QY of the WS_2_ monolayer after K doping. It should be noted that the maximum duration of BV and K doping is determined to the highest level possible within the range where the PL signal can still be distinguished from the background. In order to maximize the signal-to-noise ratio and widen the range of incident powers for evaluating the optical properties of BV- and K-doped WS_2_ monolayers, the strategy to enhance light-matter interactions can be employed. For example, the introduction of nanostructures to increase outcoupling modes will enable a broader range of doping conditions and excitation powers. Furthermore, as a follow-up study, the altered photocarrier dynamics can also be examined upon the inclusion of nanostructures or nanoparticles based on free-carrier semiconductors [30,31].

**Figure 6:**
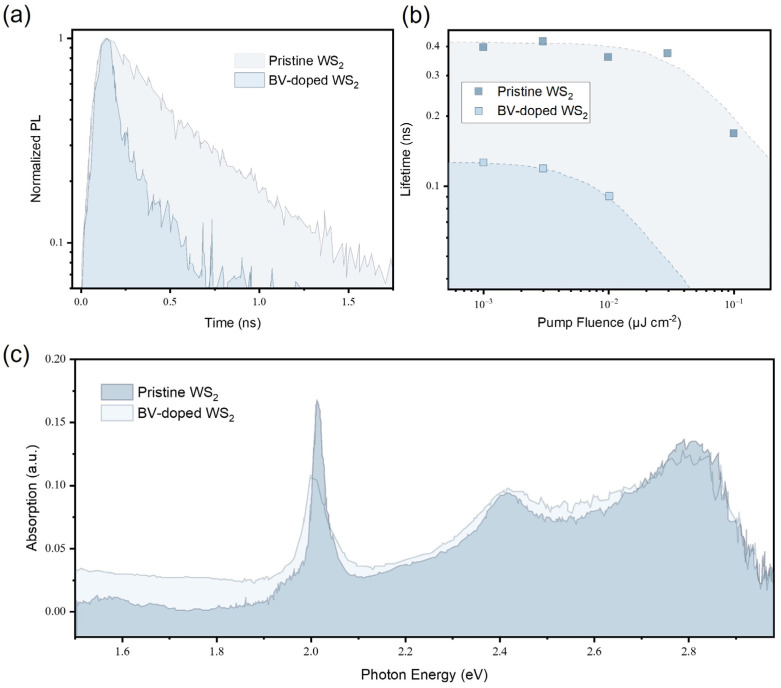
(**a**) Radiative decay of a pristine WS_2_ monolayer and a doped-WS_2_ monolayer with 1000 s of BV exposure. Time-resolved PL decay curves were acquired at a pump fluence of $3\times 10^{-3}$ μJ/cm^2^ (**b**) PL lifetime as a function of pump fluence for a pristine WS_2_ monolayer and a doped-WS_2_ monolayer with 1000 s of BV exposure. (**c**) Absorption spectra of a pristine WS_2_ monolayer and a doped-WS_2_ monolayer with 1000 s of BV exposure.

Moreover, dynamic luminescence behaviors were investigated for the WS_2_ monolayer before and after BV doping. Figure 6a presents the time-resolved PL decay of a WS_2_ monolayer to show the effect of BV doping on its recombination kinetics. Here, the radiative decay curves were fit by single exponential decay curves to extract lifetimes. As a result, the pristine WS_2_ monolayer exhibits a luminescence lifetime of ~0.4 ns, which is consistent with a previous report [32]. The lifetime of the WS_2_ monolayer then decreased to ~0.1 ns after 1000 s of BV exposure. Time-resolved PL measurements were performed with varying pump fluences, as shown in Figure 6b. At all pump fluences, it appears that BV doping shortens the lifetime of the WS_2_ monolayer, arising from the fact that the luminescence decay is mainly dictated by the trion nonradiative recombination after BV doping to transfer the high density of electrons in the monolayer. It should be noted that the lifetime becomes shorter with increasing pump fluences owing to the exciton–exciton annihilation [27]. Photocarrier dynamics can also be evaluated using transient absorption spectroscopy, which enables one to capture the ultrafast decay of excited states. However, transient absorption spectroscopy requires higher pump intensity compared to time-resolved PL spectroscopy, limiting the dynamic range of measurement for recombination kinetics [33]. Figure 6c shows the absorption spectra of the pristine WS_2_ monolayer in comparison with the WS_2_ monolayer after 1000 s of BV exposure. While there was no measurable difference in terms of shapes and resonances between the two absorption spectra, the peak near the band edge of WS_2_ shows ~30 meV redshift after BV doping. This is consistent with the PL shift induced by doping, which is associated with the trion formation from neutral excitons.

### 3.4. Luminescence Properties of the Electrically Doped 2D Monolayer Semiconductor

**Figure 7:**
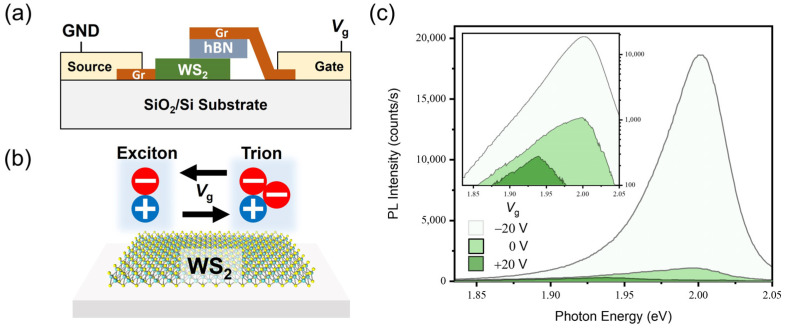
(**a**) Schematic describing the top-gated device structure. The top graphene layer acts as a gate electrode to apply electrostatic doping onto the WS_2_ monolayer, while the bottom graphene is electrically ground. (**b**) Gate voltage-dependent modulation of PL in the WS_2_ monolayer enabled by adjusting the exciton and trion density. (**c**) PL spectra of the WS_2_ monolayer with gate voltages of *V*_g_ = −20 V, 0 V, and +20 V measured at an incident power of 1 W/cm^2^.

Besides the chemical doping methods, doping can also be achieved with electrostatic approaches. We evaluated the effectiveness of electrostatic doping by fabricating and characterizing a device where a gate voltage is applied with varying generation rates. Figure 7a displays a schematic of the two-terminal capacitor device structure with the top graphene as a gate electrode and the bottom graphene as a source electrode. Through simultaneous modulation of the gate voltage and generation rate, dominant recombination pathways in monolayer semiconductors are altered. As depicted in Figure 7b, at a negative *V*_g_, electrons are taken away, and predominant recombination in the WS_2_ monolayer becomes radiative due to neutral excitons. At a positive *V*_g_, more electrons are injected into the WS_2_ monolayer, leading to an increase in nonradiative recombination from negative trions. This gate voltage-dependent PL modulation of the WS_2_ monolayer is measured and provided in Figure 7c. As the applied gate voltage is modulated from a negative to positive bias, the higher concentration of trions formed from many electrons induces the PL intensity to decrease and the redshift of the PL spectrum, which coincides with the effect of chemical n-doping, such as BV and K. Here, the incident power (P) is converted into the exciton generation rate (G) according to the equation below:

(5) $$G=\frac{\alpha P}{A\hbar \omega }$$

where α is the absorption at the excitation photon energy, A is the area of the laser spot, and ħω is the excitation photon energy. 

**Figure 8:**
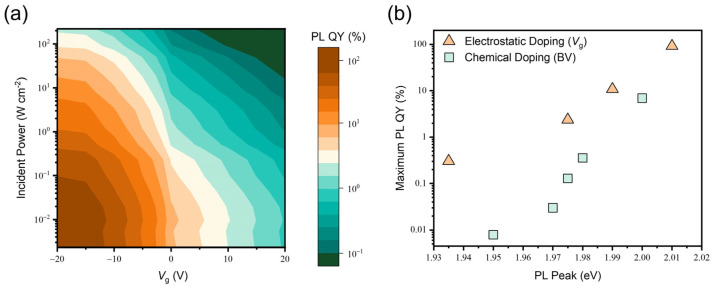
(**a**) PL QY of the WS_2_ monolayer at different gate voltages and incident powers. (**b**) The relationship between the maximum PL QY and the PL peak position of the WS_2_ monolayer after electrostatic and chemical doping.

Figure 8a shows the PL QY of the WS_2_ monolayer measured under simultaneous variation of P and $V_{g}$. In the two-terminal capacitor device configuration, the total negative charge concentration (N) is calculated by the equation:

(6) $$N=C_{OX}\frac{V_{g}-V_{th}}{q}$$

where $V_{th}$ is the threshold voltage. Since the applied gate voltages adjust the total negative charge concentration (N) and the incident powers tune the generation rate (G), the free electron concentration ($n_{e}$) in the electrically-doped WS_2_ monolayer can be extracted by numerically solving Equations (3) and (4) with substitution of the experimental parameters as well as the values for WS_2_, including $\tau_{X_{r}}=2\;ns$, $\tau_{T_{r}}=0.032\;\mu s$, $\tau_{T_{nr}}=0.1\;ns$, $T=5\times 10^{-12}\;{cm}^{2}$, and $C_{bX}=2.4\;{cm}^{2}s^{-1}$ reported in a previous work [27]. The free electron concentration of the WS_2_ monolayer device at *V*_g_ = 20 V is therefore calculated as $3.12\times 10^{12}\;{\mathrm{cm}}^{-2}$. 

The relationship between the maximum PL QY and the PL peak position of the WS_2_ monolayer under electrostatic and chemical doping is presented in Figure 8b. This result suggests that both doping methods (Electrostatic and BV) cause plenty of free electron injection followed by trion formation, leading to the dominance of nonradiative recombination. It is shown that BV doping suppresses the PL QY of the WS_2_ monolayer more significantly than electrostatic doping, curtailing the maximum PL QY by almost three orders of magnitude. This discrepancy between the two doping types originates from the fact that the free electron concentration of the BV-doped WS_2_ monolayer is higher than the electrically-doped WS_2_ monolayer. Moreover, it has been reported that the chemical doping methods (BV and K) studied in this work are known to induce degenerate doping effects, lifting the Fermi level near to the conduction band edge [16,17]. 

## 4. Conclusion

In summary, the optical properties of an electrically doped WS_2_ monolayer with surface charge transfer have been examined. BV doping and K doping both induce the PL spectra to be redshifted and the PL intensity to decrease, which is associated with the increased trion concentration. This is also supported by time-resolved PL and micro-absorption measurements. Based on the exciton and trion recombination model, the electron concentration of an electrically-doped WS_2_ monolayer was extracted. The approach we took was to numerically solve the PL QY equation which is a function of the trion nonradiative recombination rate and the exciton radiative recombination rate at different generation rates. 

In comparison with electrostatic doping, chemical doping methods such as BV and K doping cause degenerate doping in 2D TMDC monolayers. Therefore, further studies are required to unravel the correlation between the electrical parameters (electron concentration and mobility) and optical properties, especially for degenerately doped 2D monolayer semiconductors. Moreover, this work will provide opportunities for developing advanced doping technology, which can reversibly tune the exciton and trion density in 2D semiconductors. 

## Data Availability

The data presented in this work are available upon request addressed to the corresponding author.
